# The association between obstructive sleep apnoea and wound healing: a systematic review

**DOI:** 10.1007/s11325-022-02660-9

**Published:** 2022-07-28

**Authors:** Kyra Bartolo, Elizabeth A. Hill

**Affiliations:** Nuffield Department of Clinical Neurosciences, University of Oxford, Oxford, United Kingdom

**Keywords:** Obstructive sleep apnoea, Sleep breathing disorder, Hypoxia, Wound healing

## Abstract

**Purpose:**

Obstructive sleep apnoea (OSA) is a common, significantly underdiagnosed sleep-related breathing disorder, characterised by upper airway collapse and resultant intermittent hypoxia. Oxygen plays an important role in collagen synthesis and as a result in wound healing. An association between OSA and wound healing has not been clearly delineated. A systematic review was performed to understand this association.

**Methods:**

Randomised controlled trials, cohort, cross-sectional and case–control studies evaluating the relationship between OSA or OSA-related symptoms and wound healing in adult populations were searched in the systematic review using electronic databases PubMed, EMBASE and Ovid MEDLINE.

**Main results:**

A total of 11 cohort studies and 1 case–control study with a total of 58,198,463 subjects were included. Most studies suggest that patients diagnosed with OSA or who are at high risk of having OSA are more likely to suffer from wound complications. Patients with OSA have been found to be at higher risk for post-operative wound infection and wound dehiscence. Contradictory results were obtained on time to heal, with one study concluding that individuals with OSA were more likely to heal earlier when compared to patients without OSA. Quality of evidence, however, was deemed very low due to high risk of bias.

**Conclusions:**

This systematic review did identify an association between OSA and wound healing. However, due to the very low-quality evidence, further research is warranted to better characterise this association and investigate whether or not treating OSA can indeed affect wound healing.

## Introduction

Obstructive sleep apnoea (OSA) is a sleep-related breathing disorder, characterised by upper airway collapse and resultant intermittent hypoxia and sleep fragmentation [1]. It is thought to affect up to 38% of the adult population [2], that is, almost 1 billion adults aged 30–69 years worldwide [3]. Consequences of untreated OSA are multiple, impacting negatively on cardiovascular, cerebrovascular and metabolic outcomes, and increasing the incidence of occupational and road traffic accidents. These consequences are also associated with significant economic costs [4]. Eighty percent of individuals with OSA have multiple comorbidities [5].

Diabetes affects almost half a billion individuals worldwide, with prevalence continuing to rise [6]. Diabetes and OSA frequently co-exist, and a complex bi-directional relationship is proposed [7]. Diabetic foot ulcers are a serious complication of diabetes, affecting quality of life and preceding 85% of diabetes-related foot amputations [8], and are commonly seen in individuals with comorbid OSA and diabetes [7].

The prevalence of OSA is considerably higher in individuals with diabetic foot ulcers or other chronic wounds; more than half the population of patients with diabetic foot ulcers are diagnosed with moderate to severe OSA [9], whilst 57% of those with chronic wounds were found to suffer from the disorder [10]. Similar to untreated OSA, diabetic foot ulcers and failure of wound healing burden the healthcare system [8, 11].

Wound healing is a complex process influenced by multiple factors. Important steps in tissue repair include the synthesis of collagen and the conversion of fibroblasts to myofibroblasts. Both these processes are oxygen dependent [12], and intermittent hypoxia, as a result of OSA, is believed to affect tissue survival and wound healing both directly, as well as through sympathetic nervous system overactivity, inflammation and oxidative stress [10]. However, this association has not been clearly delineated.

Wounds that fail to proceed through the normal phases of healing in an orderly and timely manner develop into chronic wounds [13]. Through the understanding of the different stages of wound healing, one can screen for specific predictors of tissue repair and target therapy accordingly in order to induce earlier healing and avoid formation of chronic wounds. Several observational studies have sought to identify the link between OSA and wound healing in adult populations. Therefore, a systematic review was carried out to characterise this possible association. The objective of this review was to evaluate the effect of OSA on wound healing.

## Criteria for considering studies for this review

The literature was reviewed for all randomised controlled trials and observational studies published in the English language, including cohort, cross-sectional and case–control studies evaluating the relationship between OSA or OSA-related symptoms and wound healing. Case reports and case series were excluded from the study.

Included studies had to be focused on human adult populations aged 18 years and older. No restriction was imposed in terms of inclusion or exclusion criteria, for example pregnancy.

Required outcome measures included:

- Post-operative wound healing—time to heal and occurrence of wound complications.
- Skin ulcer healing—time to heal.

## Search methods for identification of studies

The criteria of the Preferred Reporting Items for Systematic Review and Meta-Analyses (PRISMA) were followed [14]. A systematic search of the electronic databases PubMed, EMBASE and Ovid MEDLINE was performed up to January 6, 2021. The search strategies used a combination of keywords to describe two concepts: OSA and wound healing. The following terms were used: “Obstructive Sleep Apn*” or OSA or OSAS or OSAHS or “Sleep Apn* Hypopn* Syndrome” or “Upper Airway Resistance Syndrome” or “Sleep Disordered Breathing” and wound or “wound healing”.

As the review was part of a programme of academic study, the protocol was not prospectively registered by the author prior to commencement.

The titles and abstracts were checked against the eligibility criteria and full-text versions of potentially eligible publications were reviewed in order to determine suitability for inclusion. The reference lists from the included studies and relevant review articles were also checked for potential publications not retrieved by the electronic search that might meet eligibility criteria. Full reports of review articles were also retrieved and checked for any relevant citations.

## Data extraction

Data were extracted from all eligible studies using a standardised extraction form. The data included were first author’s name, publication year, study design, population, sample size, wound type, wound parameter studied, method of OSA diagnosis, mean age of patients, percentage of male patients and number of subjects with OSA. The data were recorded in a pre-formatted Excel spreadsheet.

## Quality assessment

The Newcastle–Ottawa Scale (NOS) was modified and used to assess the methodological quality of the selected non-randomised studies [15].

The NOS was used to appraise the following characteristics:

- Selection (4 items): representativeness of exposed cohort; representativeness of non-exposed cohort; ascertainment of exposure and demonstration that outcome of interest was not present at start of study.
- Comparability (2 items): adjusting for the most important risk factors and study control for at least 3 additional risk factors.
- Outcome (3 items): assessment of outcome; was follow-up long enough for outcomes to occur; adequacy of follow-up of cohorts. (Used in Cohort studies)
- Exposure: ascertainment of exposure; same method of ascertainment for cases and controls. (Used in case–control studies)

The quality of each study was indicated by a star rating system. A maximum of nine stars could be awarded, with a maximum of one star for each numbered item within the selection and outcome or exposure categories and a maximum of two stars for comparability. The following thresholds were then used to convert the Newcastle–Ottawa scales to AHRQ (Agency of Healthcare Research and Quality) standards (good, fair and poor): good quality: 3 or 4 stars in selection domain AND 1 or 2 stars in comparability domain AND 2 or 3 stars in outcome/exposure domain; fair quality: 2 stars in selection domain AND 1 or 2 stars in comparability domain AND 2 or 3 stars in outcome/exposure domain; poor quality: 0 or 1 star in selection domain OR 0 stars in comparability domain OR 0 or 1 stars in outcome/exposure domain.

The Newcastle–Ottawa Scale was adapted for assessment of risk of bias in the included studies. For the purpose of assessment of selection bias, a cohort which was identified as truly or somewhat representative of the average adult population was one which had no or limited risk factors for poor wound healing. Cohorts which were identified as being at risk for poor wound healing even prior to the studied exposure were considered at high risk of selection bias.

In order to assess for selection bias through ascertainment of exposure, a star was awarded when participants were assessed for OSA by the gold standard test—polysomnography or equivalent. In this manner, a clear diagnosis of OSA with an apnoea hypopnoea index (AHI) is obtained. In addition, all the study participants must have undergone the investigation in order to confirm the presence or absence of a diagnosis of OSA. Obtaining a diagnosis from medical records alone was considered as reason for selection bias.

When assessing the comparability of cohorts on the basis of the design or analysis, one star was awarded when the study controlled for age alone. Gender was not considered in this case since half the studies were carried out in a female population. An additional star was awarded when the study controlled for at least 3 additional risk factors.

When assessing for outcomes bias, duration of follow-up was considered satisfactory, depending on the particular outcome studied.

Since this systematic review aims to understand the association between OSA and wound healing, the outcome measures used included time to heal and wound complications such as wound infection and dehiscence. The quality of a body of evidence for a specific outcome was based on the Grading of Recommendations Assessment, Development and Evaluation (GRADE). Criteria for evaluating the quality of evidence included limitations in the design and implementation, indirectness of evidence, unexplained heterogeneity or inconsistency of results, imprecision of results and high probability of publication bias. GRADE is used to determine the level of confidence that the estimate of the effect is correct [16].

## Results

**Fig. 1:**
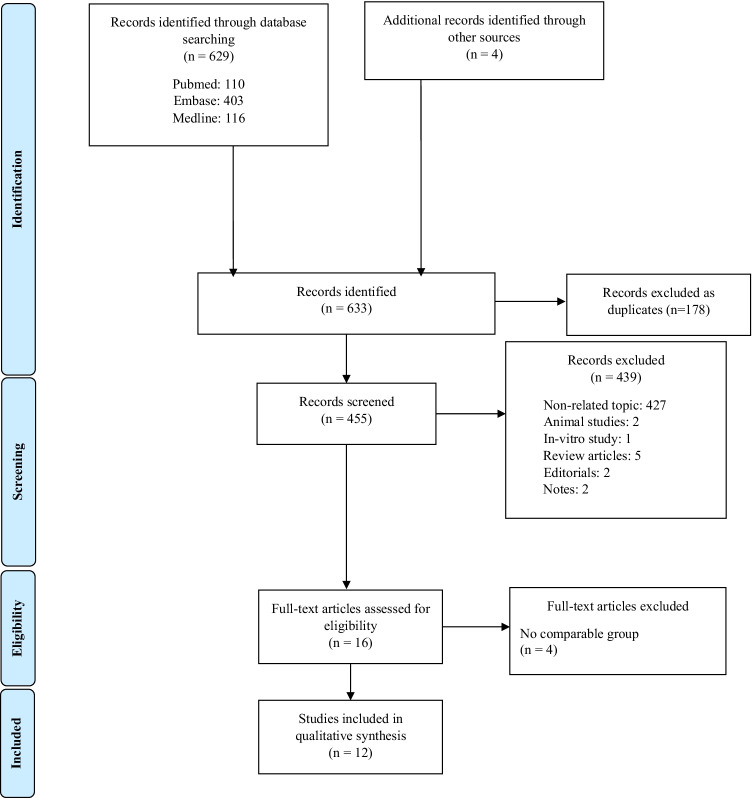
PRISMA flow diagram

The PRISMA flowchart of study selection is shown in Fig. 1. The database searches yielded 451 records after removal of duplicates. After screening titles and abstracts, 439 records were excluded. Of these reports, 427 focused on a non-related topic, 2 were animal studies, 1 in vitro study and 9 included review articles, editorials and notes. Four relevant studies were identified in a review article and from the reference list of an included article. After assessing the remaining 16 full-text articles, 4 were excluded because there was no comparable group. Ultimately, twelve studies with a total of over 57 million participants were included. The general characteristics and patient demographics are listed in Tables 1, 2, 3 and 4.

**Table 1:** Characteristics of included studies

Author, year	Study design	Population	Participants	Exposure – diagnosis of OSA	Type of wound	Wound outcome	Age (mean ± SD)	Male gender (*N*, %)	OSA (*N*, %)
Weingarten et al., 2011	Retrospective cohort	USA (Minnesota)	797	In-patient overnight polysomnography	Surgical incision –laparoscopic or open bariatric surgery	Wound infection, wound dehiscence	46.6	206 (25.8)	618 (77.5)
Andrews et al., 2012	Retrospective cohort	USA (Minnesota)	307	As documented on electronic note retrieval database	Partial-foot amputation site	Time to heal	65.5 ± 14.8	213 (69.4)	25 (7.7)
D’Apuzzo et al., 2012	Retrospective national cohort	USA (Virginia)	258,453	ICD-9-CM OSA code as documented in the Nationwide Inpatient Sample database	Surgical incision – Revision Joint Arthroplasty	Wound dehiscence, haematoma, seroma	66.5	108,292 (41.9)	16,608 (6.4)
Louis et al., 2012	Prospective cohort	USA (Ohio)	175	In-home portable polysomnography using ARES unicorder 5.2 (B-Alert, Carlsbad, CA)	Surgical incision – C-section	Post-operative wound complications – not defined	OSA: 27.3 ± 5.9 No OSA:30 ± 6.44	0	27 (15.4)
Louis et al., 2014	Retrospective national cohort	USA	55,781,965	ICD-9-CM OSA code as documented in the Nationwide Inpatient Sample database	Surgical incision – C-section	Post-operative wound complications – not defined	Not available	0	3 per 10,000 (0.03)
Fortis et al., 2015	Retrospective cohort	USA (Minnesota)	110	As documented in the anaesthesia pre-operative records	Surgical incision—colectomy	Wound infection	61.5 ± 11.5	60, (54.5)	42 (38.1)
Spence et al., 2016	Retrospective cohort	USA (San Diego)	305,001	ICD-9-CM OSA code as documented in the Military Health System Management Analysis and Reporting Tool	Surgical Incision – C-section	Post-operative wound complications – not defined	Not available	0	266 (0.09)
Bamgbade et al., 2016	Prospective cohort	Canada (Vancouver)	160	WatchPAT 200 (Itamar Medical, Caesarea, Israel)	Surgical Incision for gynaecologic oncology abdominal surgery	Wound infection, wound dehiscence	58 ± 16	0	80 (50)
Bourjeily et al., 2017	Retrospective national cohort	USA (Providence)	1,577,632	ICD-9-CM OSA code as documented in the National Perinatal Information Centre	Surgical incision – C-section	Post-operative wound complications – not defined	Not available	0	1963 (0.12)
Maltese et al., 2018	Prospective cohort	UK (London)	94	STOP-BANG score	Diabetes-related foot ulcers	Diabetic foot ulcer persistence ± re-occurrence	Not available	65 (69)	NA
Dang et al., 2019	Case–control	USA & Canada	Cases: 1291 Controls: 272,346	As documented on patient data registry	Bariatric surgery incision	Wound infection	44.6 ± 12	57,305 (20.1)	103,651 (37.9)
Ozdilekcan et al., 2020	Retrospective cohort	Turkey	132	Berlin questionnaire, STOP-BANG score, ESS	Mastectomy/breast conserving surgery wound	Wound complications: seroma, haematoma, wound infection, delayed wound healing, incisional wound dehiscence and nipple necrosis	54.6 ± 11.6	0	NA

**Table 2:** A detailed Newcastle–Ottawa Scale of each included cohort study

	Selection				Comparability		Outcome			
Study	Representativeness of exposed cohort	Selection of non-exposed cohort	Ascertainment of exposure	Demonstration that outcome of interest was not present at start of study	Adjust for the most important risk factors	Study controls for at least 3 additional risk factors	Assessment of outcome	Follow-up length	Loss to follow-up rate	Total Quality Score
Weingarten, 2011	-	*	*	*	*	*	*	*	-	7 Good
Andrews, 2012	-	*	-	*	*	*	*	*	*	7 Fair
D’Apuzzo, 2012	*	*	*	*	*	*	*	-	-	6 Poor
Louis, 2012	-	*	-	*	*	-	*	*	-	5 Fair
Louis, 2014	-	*	-	*	*	*	*	-	-	5 Poor
Fortis, 2015	*	*	-	*	*	*	*	-	-	6 Poor
Spence, 2016	-	*	*	-	*	*	*	-	-	5 Poor
Bamgbade, 2017	-	*	*	*	-	-	*	-	*	5 Poor
Bourjeily, 2017	-	*	-	*	*	*	*	-	-	5 Poor
Maltese, 2018	-	*	-	*	*	*	*	-	*	6 Poor
Ozdilekcan, 2020	-	*	-	*	*	*	*	-	*	6 Fair

**Table 3:** A detailed Newcastle–Ottawa Scale of included case–control study

	Selection				Comparability		Exposure		
Study	Is the case definition adequate?	Representativeness of cases	Selection of controls	Definition of controls	Adjust for the most important risk factors	Study controls for at least 3 additional risk factors	Ascertainment of exposure	Same method of ascertainment of cases/controls	Total Quality Score
Dang, 2019	-	*	-	*	*	*	-	*	5

**Table 4:** Summary of findings

Outcome	Author, year	Number of participants	Effect size index	Effect size (with 95% confidence intervals)	Quality of the evidence (GRADE)
Post-operative wound complications	Louis. 2012	56,882,940 (5 studies)	Adjusted OR	3.44 (0.7–16.93)	Very low
	Louis, 2014		Adjusted OR	1.89 (1.53–2.34)	
	Spence, 2016		Adjusted OR	2.47 (0.6–10.14)	
	Bourjeily, 2017		Adjusted OR	1.77 (1.24–2.54)	
	Ozdilekcan, 2020		HR (STOP-BANG questionnaire high risk)	2.18 (1.44–2.96)	
			HR (BERLIN questionnaire high risk)	1.96 (1.22–2.43)	
Surgical site infection	Weingarten, 2011	533,157(5 studies)	OR (open surgery)	1.14 (0.96–1.74)	Very low
			OR (laparoscopic surgery)	1.355 (1.06–3.47)	
	D’Apuzzo, 2012		Adjusted OR	1.25 (1.03–1.51)	
	Fortis, 2015		Adjusted OR	3.98 (1.29–12.27)	
	Bamgbade, 2017		OR	1.82 (0.81–5.75)	
	Dang, 2019		OR	1.576 (1.46–1.76)	
Wound dehiscence	Weingarten, 2011	259,410 (3 studies)	OR (open surgery)	1.697 (1.52–3.205)	Very low
			OR (laparoscopic surgery)	1.097 (0.976–9.381)	
	D’Apuzzo, 2012		Adjusted OR	1.25 (1.03–1.51)	
	Bamgbade, 2016		OR	3.05 (0.38–97.38)	
Effect of OSA/risk on duration of healing	Andrews, 2012	401 (2 studies)	OR	5.6 (1.4–22.7)	Very low
	Maltese, 2018		Adjusted Relative Risk	2.67 (1.14–4.03)	

### Country

Nine of the twelve studies were conducted in the USA. The other three studies were conducted in Turkey, Canada and the UK.

### Study design

Eleven cohort studies and one case–control study investigated the association of wound healing or wound complications with OSA.

### Participants

A total of 58,198,463 subjects were identified. A very small proportion of participants were male (0.29%) as six of the studies identified wound healing as an outcome in populations which underwent breast surgery, caesarean sections and gynaecological oncology abdominal surgery.

### Defining the exposure—diagnosis of obstructive sleep apnoea

Whilst 6 of the studies used electronic patient databases for the identification of subjects diagnosed with OSA, only three cohort studies sought to achieve a formal diagnosis of OSA using in-patient overnight polysomnography, WatchPAT 200 (Itamar Medical, Caesarea, Israel) and ARES unicorder 5.2 (B-Alert, Carlsbad, CA). Ozdilekcan et al. assessed risk of exposure by utilising the STOP-BANG score and Berlin questionnaire, screening tools for OSA as well as the Epworth Sleepiness Scale, a questionnaire measuring daytime sleepiness [17]. Maltese et al. utilised the STOP-BANG score alone [18]. Both studies assessed the association between wound healing and risk of exposure to OSA rather than a confirmed diagnosis of the sleep breathing disorder.

### Outcome of interest

The studied wounds varied widely. Whilst only a single study focused on diabetic foot ulcers, the rest of the studies looked at surgical wounds in a number of different surgical sites. These included wounds at amputation sites, abdominal and pelvic wounds, mastectomy and breast conserving surgery wounds as well as arthroplasty incisions. Reported wound parameters identified time to heal and the occurrence of wound complications. The latter included wound infection and wound dehiscence.

### Synthesis of results

In all the four cohort studies which looked at the impact of OSA on pregnancy-related outcomes, an outcome of which was undefined post-operative wound complications, adjusted odds ratios were found to show an association between OSA and wound healing. Whilst Louis et al. [19] and Bourjeily [20] reported adjusted odds ratios with almost double the risk of wound complications in patients diagnosed with OSA; Louis [21] and Spence [22] reported odds ratios with wide confidence intervals spanning the value of 1.

Calculated odds ratios for the impact of OSA with wound dehiscence and surgical site infection of incision wounds for gynaecologic oncology abdominal surgery showed no association [23].

Weingarten [24] and Dang [25] both looked at the association between OSA and post-operative bariatric surgery wound healing. Both authors reported a clear association between OSA and laparoscopic surgical site infection. No relationship between OSA and open surgical site infection was however identified in Weingarten’s study. On the other hand, an increased incidence of wound dehiscence was reported in OSA patients who underwent open surgery.

Fortis and colleagues [26] identified OSA as a predictor of surgical site infection following colectomy. After controlling for confounding variables, D’Apuzzo [27] identified OSA as a risk factor for wound infection and wound dehiscence in patients having undergone revision joint arthroplasty.

Ozdilekcan et al. [17] and Maltese et al. [18] used validated surveys to study the association between risks for OSA and wound healing. Being in medium high risk on STOP-BANG questionnaire or being high risk on Berlin questionnaire was found to be independent statistically significant risk factors for wound healing complications in breast cancer patients. Wound complications included both minor complications such as seroma, haematoma, wound infection and delayed wound healing, as well as major complications such as incisional wound dehiscence and nipple necrosis. Similarly, Maltese et al. [18] reported an association between risk for OSA as defined by an elevated STOP-BANG score and poor diabetic foot ulcer healing in both univariate and multivariate analyses.

Whilst most studies concluded that the impact of OSA on wound healing is a deleterious one, results from Andrews et al. [28] on the impact of OSA on amputation site healing reported that patients with OSA were significantly more likely to heal within 3 months when compared to patients without OSA.

## Discussion

This is the first systematic review to evaluate the effect of OSA on wound healing and, as such, provides a more robust analysis than the results of individual studies.

### Summary of main results

The term wound healing is broad and encompasses a variety of outcomes. Through this systematic review, studied outcomes included post-operative wound complications, surgical site infection, wound dehiscence and duration of healing.

Although OSA was identified as a risk factor for post-operative wound complications, data were not consistent throughout all studies. Surgical site infections occurred more commonly in patients diagnosed with OSA; however, this was not evident in all studies. No clear distinction between risks of infection in laparoscopic or open surgery was noted. OSA was found to play a role in wound dehiscence, with a higher risk of wound dehiscence in open surgery when compared to laparoscopic surgery. The effect of OSA on duration of healing showed conflicting evidence. Whilst a high risk for OSA was associated with an increased relative risk of poor diabetic foot ulcer healing, patients with a diagnosis of OSA who underwent surgery for amputation were significantly more likely to heal earlier when compared to patients with no OSA.

### Overall completeness and applicability of evidence

In this systematic review, the term wound was used to encompass both post-operative wounds and ulceration. The location of the studied wounds varied. In this way, the effect of OSA on wound healing could be extrapolated to different wound types and locations. Although a diagnosis of OSA was deemed sufficient, knowledge of the severity of OSA in the study participants would have led to greater completeness of evidence.

The selected studies did not provide information on whether or not patients were given advice on standard wound care. Most studies reported wound complications which included infection and dehiscence rather than reporting parameters of wound healing, such as wound size. Such relevant outcomes were not investigated.

Through the use of airflow to create a positive pressure in the oropharynx or laryngopharynx, CPAP therapy is considered the gold standard treatment of OSA [29]. By obliterating the chronic intermittent hypoxia, CPAP therapy nullifies the difference between patients who suffer from OSA and those who do not. Lack of data regarding whether individuals with OSA were receiving treatment with CPAP therapy or not limits our applicability of evidence and invalidates conclusions drawn.

### Quality of evidence

#### Limitations in the design and implementation—risk of bias

Risk of bias was assessed using the Newcastle–Ottawa Scale. Several limitations in the design and implementation were identified. Only 2 of the 11 cohort studies were identified as having selected a cohort which was truly or somewhat representative of the average adult population with OSA. Where the selected population had additional exposures, which increased risk to the studied outcome, they were considered to be at high risk of selection bias. Such populations included patients diagnosed with diabetes, pregnant and post-partum women, patients undergoing bariatric surgery, surgical amputation, oncology patients undergoing gynaecological abdominal surgery or breast surgery. Age, diabetes, malignancy and pregnancy are all known to affect wound healing and may thus act as confounding factors to the outcome [11]. Patients referred for bariatric surgery were more likely to be obese, with a large part of the cohort being classified as super-obese. All studies enrolled the non-exposed cohort from the same community as the exposed cohort.

Three cohort studies were awarded a star for confirming the presence or absence of exposure of the whole studied population. Weingarten et al. [24] studied a population referred for bariatric surgery, who had all been referred for assessment of OSA using in-patient overnight polysomnography prior to surgery. Bamgbade et al. [23] assessed each participant using WatchPAT 200 (Itamar Medical, Caesarea, Israel), a device which diagnoses OSA based on peripheral arterial tonometry. Such a device is considered to provide a reliable signal and is included as an approved option for home sleep assessment of OSA as per the AASM scoring manual [30]. Louis and colleagues [21] used ARES unicorder 5.2 (B-Alert, Carlsbad, CA) for detecting OSA in pregnant women. The Apnoea Risk Evaluation System (ARES) home sleep test uses a device which incorporates blood oxygen saturation, pulse rate, airflow and snoring levels to confirm or exclude the presence of OSA. ARES unicorder 5.2 (B-Alert, Carlsbad, CA) was found to demonstrate consistently high sensitivity and specificity for both in-laboratory and in-home recordings [31]. Of the included studies, only the above three were able to provide a diagnosis and severity of OSA based on AHI. In the retrospective national cohorts, exposure was based on records documented in nationwide databases using ICD-9-CM OSA codes. Such studies were unable to provide severity of the disease. Retrospective coded data is associated with limitations based on the risk of inconsistencies in coding and documentation. This leads to a potential diagnostic variability of OSA. One cannot assume that patients receiving an ICD-9 code of OSA were diagnosed using standard OSA diagnostic criteria. In such cohort studies, undercoding is a further limitation which could introduce ascertainment bias. In these studies, individuals who have not undergone investigation for OSA are assumed to not have the exposure. Also, the use of discharge data for patient databases may potentially introduce reporting bias since physicians are more likely to document a diagnosis of OSA in patients who have suffered surgical complications. Treatment of OSA with CPAP therapy may also impact the interpretation of results. Only Andrews et al. [28] included data on patients who were on treatment therapy. Maltese et al. [18] and Ozdilekcan et al. [17] studied the impact of risk of exposure rather than the exposure itself. Through the use of the STOP-BANG questionnaire, Berlin questionnaire and Epworth Sleepiness Scale, participants were stratified as being either at low or high risk for OSA. Although these risk assessment tools may show high sensitivity, specificity and positive predictive values for identifying OSA, studies looking at risk of exposure were considered to have high selection bias since the presence or absence of OSA was not confirmed.

Considering that this systematic review looks at wound healing as an outcome of interest, the fact that all but one of the studies assessed post-operative wound outcome demonstrated that the outcome of interest was not present at the start of the study. Maltese et al. [18], who studied diabetic foot ulcer healing prospectively, included a cohort of patients who had already been attending the diabetic foot clinic service for at least 2 months. Since their primary outcome was poor healing of diabetic foot ulcer, defined as diabetic foot ulcer persistence and or diabetic foot ulcer re-occurrence at 12 months, any ulcer which had been present for several months prior to inclusion in the study may have been falsely classified as healed despite lasting for longer than a total of the 12-month duration.

When assessing the comparability of cohorts on the basis of the design or analysis, confounding factors must be adjusted for in the analysis. Any patient characteristic known to influence wound healing should be accounted for. Whilst all studies controlled for age, all but two controlled for at least three additional covariates. Six of the studies were based on female individuals, and thus, the gender was not chosen as a fundamental risk factor to be controlled for. Risk factors which were commonly accounted for in multivariable logistic regression analysis included race, body mass index, smoking status and comorbidities such as diabetes, hypertension, peripheral vascular disease and hyperlipidaemia, and in cases of post-operative wounds, the surgical procedure and operative time were accounted for. Chronic steroid use and immunosuppressant use, both factors known to delay wound healing, were only accounted for in the case–control study by Dang et al. [25].

In assessment of outcome, considering that the majority of included studies are retrospective cohort studies, reference to medical records was considered sufficient to satisfy the requirement for wound healing parameters or complications. Apart from how and where details on outcome were obtained, it is also important to identify the quality of outcome parameters assessed. Due to the fact that the national cohort studies assessed the impact of OSA on several post-operative outcomes, very limited data was available on wound healing. The only documented parameter was the presence of post-operative wound complications. These complications were not defined. On requesting additional information from one of the authors, she was unable to provide granular details about the actual complications, as these were not available since outcomes were based on ICD-9 codes rather than chart-level detail (personal communication). It was only in the prospective study on diabetic foot ulcers that independent blind assessment was carried out. Most studies lacked clear criteria for wound healing, non-healing and complications. Although wound healing itself is many a time based on subjective assessment, clear criteria for the definition of a healed wound or wound complication is considered essential. Whilst Ozdilekcan [17] clearly defined wound complications and Dang et al. [25] described criteria for the diagnosis of superficial and deep incisional surgical site infections, other authors failed to outline a clear definition of their outcome parameters.

Only four of the cohort studies provided data on the duration of the follow-up. Since these three studies assessed different wounds, no standard follow-up length was chosen to assess whether the duration of the follow-up was considered satisfactory or not. Thirty days for bariatric surgery wound assessment, 3 months of follow-up of amputation site healing and 6 weeks for post-partum wound complications were all considered satisfactory follow-up lengths based on related studies. In a study focused on amputation wound healing, Baumfeld and colleagues concluded that wound healing lasted an average of 35 days in a population of diabetic patients [32]. In the study by Maltese et al. [18], members of the diabetes foot team assessed all participants at 12 months. Where in the included studies, no duration of follow-up was made available, no star was awarded and an additional option of no statement on duration of follow-up was added to the NOS criteria.

The majority of cohort studies failed to include details on the adequacy of follow-up of cohorts. Whilst in three of the studies [17, 18, 28] all subjects were accounted for, in the study by Bamgbade et al. [23], since subjects followed up were greater than 90% of the original population, this was not considered to impact outcome bias. Contrary to this, Louis et al. [21] reported a follow-up rate which was less than 90% and as a result increased outcome bias.

### Quality of evidence

#### Indirectness, heterogeneity and imprecision of results

The initial quality of evidence for all outcomes of interest was assessed as being low, as all included studies were observational studies. Apart from the high risk of bias described above, indirectness of evidence was also identified and noted to play a role in downgrading our level of confidence that the calculated estimate of the effect is correct. Due to the studied populations being varied with multiple comorbidities, mostly not representative of the general population, they were considered to be sufficiently different to cause substantial difference in the magnitude of effect. Other than indirectness due to population differences, lack of similarity was also noted in outcome measures. The absence of clear definitions for outcome parameters such as the diagnostic criteria for incisional surgical site infections leads to indirectness in the surrogate outcomes.

Both clinical and methodological heterogeneity are identified in the studies put together in this systematic review. Variability identified in the participants, outcomes and the study design will lead to the true effect being different in the different chosen studies. For each studied outcome, a number of studies had relative effect sizes with wide confidence intervals implying imprecision of results.

When working on observational studies, publication bias may pose a more significant problem, when compared to randomised studies. The likelihood of a negative observational study being published may be lower than for a positive one, thus leading to a high probability of publication bias.

Due to high risk of bias, indirectness of evidence, heterogeneity, imprecision of results and high probability of publication bias, the quality of the evidence for each studied outcome was downgraded from low to very low.

#### Potential biases in the review process

As any other systematic review, this too comes with some limitations, which should be considered when interpreting the results. Firstly, the review was conducted as part of an academic programme of study, and as such, the protocol was not prospectively registered by the author prior to commencement. Although the search strategy sought to be as comprehensive as possible, some studies may have been missed. Excluding related literature published in languages other than English may have introduced a language bias. The majority of cases were observational retrospective cohort studies, which do not provide the same level of evidence as experimental studies. Exclusion criteria may have been more stringent in criteria for diagnosis of exposure and outcome measures. The variety in definition and severity of exposure may compromise the comparability of studies. Different means of diagnosing OSA or determining the risk of OSA impacts the reliability of this review. Another limitation is the fact that outcome measures varied among studies.

## Conclusions

To date, this is the first review to systematically evaluate the relationship between OSA and wound healing.

Wound complications and chronic wounds continue to be a challenging problem. The morbidity, mortality and costs associated with chronic wounds, including diabetic foot ulcers, represent a considerable global healthcare burden [8, 11]. This major medical and financial burden highlights the need to implement novel ways to prevent and treat these wounds. Given the complex relationship between OSA and diabetes and a higher population prevalence of OSA in patients with chronic wounds, it is clear that OSA plays a role in the pathophysiology of poor wound healing. Despite very low-quality evidence in this systematic review, OSA is seen to affect the process of wound healing and increase the risk of some wound complications. Diagnosing and treating OSA in this population of patients may prove to be a truly cost-effective measure, though further research is required.

To date, no randomised controlled trial has been performed to study the effect of OSA and CPAP therapy on wound healing. Future research should look at individuals who have been diagnosed with OSA using gold standard testing, such as polysomnography, and where the use of CPAP therapy is known. Mechanistic studies examining the complex pathophysiological relationship between OSA, diabetes and wound healing are required. Through randomised controlled trials studying the effect of CPAP therapy, which obliterates the apnoeas in OSA and thus avoids intermittent hypoxia which is implicated in the process of wound healing, we can better understand the influence of OSA on such an important process, with the aim of reducing wound complications and delays in wound healing. This in turn may improve the quality of life for patients and reduce economic and societal burden worldwide.

## Data Availability

As this research is a systematic review, all data was extracted from the included studies. All data generated or analysed during this study are included in this published article.
